# Redox Status Response of Physical Exercise Training in Women with Breast Cancer during Trastuzumab Therapy

**DOI:** 10.3390/healthcare10102039

**Published:** 2022-10-15

**Authors:** Katarzyna Hojan, Karolina Gerreth, Danuta Procyk, Krystian Mania, Anna Zalewska, Mateusz Maciejczyk

**Affiliations:** 1 Department of Rehabilitation, Greater Poland Cancer Centre, 15 Garbary St., 61-866 Poznan, Poland; 2 Department of Occupational Therapy, Poznan University of Medical Sciences, 6 Swiecickiego St., 60-781 Poznan, Poland; 3 Department of Risk Group Dentistry, Chair of Pediatric Dentistry, Poznan University of Medical Sciences, 70 Bukowska St., 60-812 Poznan, Poland; 4 Central Labolatory, Greater Poland Cancer Centre, 15 Garbary St., 61-866 Poznan, Poland; 5 Greater Poland Provincial Hospital, 9-14 Juraszow St., 60-479 Poznan, Poland; 6 Experimental Dentistry Laboratory, Medical University of Bialystok, 24A Marii Sklodowskiej-Curie St., 15-276 Bialystok, Poland; 7 Department of Hygiene, Epidemiology and Ergonomics, Medical University of Bialystok, 2C Adama Mickiewicza St., 15-022 Bialystok, Poland

**Keywords:** cancer, physical activity, rehabilitation, redox status, oxidative stress, antioxidants, oncology

## Abstract

Trastuzumab is indicated in the adjuvant setting for the early and intermediate stages of breast cancer (BC) positive for epidermal growth factor receptor 2 (HER2). Although HER2 in BC patients tends to disrupt pro-oxidant and inflammatory signaling, the influence of trastuzumab in modulating this process remains unknown. Due to the absence of any chemotherapeutic or chemoprophylactic agents for trastuzumab-induced side effects, this study investigated the potential role of regular physical exercise in modulating the antioxidant defenses, oxidative stress, and nitrosative damage in BC patients during trastuzumab treatment. Aim: The study aimed to analyze the relationship between regular physical activity and the redox status in women with BC during trastuzumab therapy. Materials and methods: We observed 50 BC patients during trastuzumab therapy in two groups: one that undertook moderately intensive supervised physical exercises, and a second that performed physical activity according to the recommendations for cancer patients, along with a third (control) group of healthy women. Results: The antioxidant enzyme and non-enzymatic antioxidant activities were significantly higher in the exercised group compared with the other participants. The concentrations of lipid and protein oxidative damage and nitrosative stress products were significantly higher in both BC groups than in the healthy controls. Conclusions: Trastuzumab treatment stimulates a redox response in BC patients. The results highlight the oxidative imbalance in parallel with regular physical training in women with BC during trastuzumab therapy. Further studies are needed to analyze different intensities and levels of physical training in women with BC during trastuzumab treatment.

## 1. Introduction 

Breast cancer (BC) is the most common cancer in women worldwide [1]. Although BC is primarily treated surgically, biological therapy is also often used. Trastuzumab is a monoclonal antibody that binds specifically to the extracellular domain of the epidermal growth factor receptor 2 (HER2) [2]. HER2 is found to be over expressed in 25 to 30% of BC patients [2]. HER2-positive tumors grow faster, are more resistant to standard chemotherapy, and are associated with recurrence [2,3]. Therefore, trastuzumab is indicated in the adjuvant setting for the early and intermediate stages (I–III) of BC and metastatic BC [2]. Trastuzumab is generally well-tolerated, with mild and primarily manageable side effects [2,3,4,5]. One of the significant side effects is a reduction in the left ventricular ejection fraction (LVEF), leading to advanced congestive heart failure [3,4,5,6]. The mechanism of this side effect is not entirely understood, but trastuzumab blocks the physiological action of HER2 on cardiomyocytes [7,8]. In the absence of HER2 function, cardiomyocytes cannot activate survival pathways, resulting in the overproduction of reactive oxygen species (ROS), mitochondrial abnormalities, and cardiac dysfunction [7,8]. The advanced stages of BC are also associated with the loss of skeletal muscle mass, leading to functional impairment and a high risk of inactivity, increasing the risk of heart failure. 

Trastuzumab has been reported to suppress autophagy by activating Erk/mTOR/Ulk 1 signaling, resulting in mitochondrial dysfunction and cardiac oxidative stress [3,4,8,9]. However, there are no effective therapeutic factors that could prevent the development of this unwanted effect of trastuzumab without compromising its efficiency. Although HER2 patients naturally exhibit disruption in pro-oxidant and inflammatory signaling, the influence of trastuzumab in modulating this process remains unknown. The exact effect of trastuzumab on systemic redox homeostasis is also unclear. In particular, little is known about its impact on nitrosative stress, which mediates, e.g., cardiovascular damage.

Numerous studies have documented how disturbances to redox homeostasis are associated with carcinogenesis, tumor progression, and therapeutic efficacy, including that for BC therapy [9,10,11,12,13]. Therapeutic strategies that modulate ROS production may be aimed at cancer prevention but may also complement anti-tumor therapy. Due to the absence of any chemotherapeutic or chemoprophylactic agents for trastuzumab-induced side effects, this study investigated the potential role of regular physical exercise in modulating the antioxidant defenses, oxidative stress, and nitrosative damage in BC patients during trastuzumab therapy. Indeed, physical activity has a significant impact on human health. According to Lalonde’s concept of health fields, an individual’s lifestyle, including their physical activity, contributes to at least 50% of their health [14,15,16]. It is believed that physical exercise and rehabilitation during oncological treatment greatly impact the quality of life. They improve physical and mental wellbeing by maintaining muscle tone, have a beneficial effect on the cardiorespiratory system, and reduce chronic fatigue and pain. Experimental studies highlighted that regular vigorous exercise could reduce tumor growth by driving changes to the immune and endocrine systems, angiogenesis, inflammatory states, and redox homeostasis [17]. The effects of trastuzumab on systemic redox homeostasis remain unknown, similar to the impact of physical exercise on oxidative and nitrosative stress in BC-treated patients; therefore, the present study aimed to analyze the relationship between regular physical activity and the redox status in women with BC during trastuzumab therapy. 

## 2. Materials and Methods

### 2.1. Study Design

This was a prospective clinical trial. The Bioethics Committee at Poznan University of Medical Sciences approved the study protocol (no. 785/14). After a detailed explanation of the purpose of the study and presentation of possible risks, all the persons involved agreed in writing to participate in the experiment. All the patients participating in the study had to obtain an oncologist’s and cardiologist’s approval. Additionally, a medical doctor rehabilitation specialist provided a detailed explanation of the study’s physical exercise program to the patients and then obtained their voluntary written informed consent to participate in this trial.

### 2.2. Study Setting and Participants

The study was performed at a cancer hospital in western Poland. Screening for BC patients positive for the HER2 receptor, performed at the hospital, was used to identify prospective study participants. Finally, 50 women with BC undergoing trastuzumab treatment were selected to study observations (to BC group with supervised physical training and additionally to BC group with usual daily activity) according to the study criteria.

The inclusion criteria were as follows: women with BC with positivity for the HER2 receptor confirmed histologically after chemotherapy, aged 18–75 years, in good general health according to European Cooperative Oncology Group (ECOG) performance status 0–2, with good heart function and LVEF above 60%, and normal functioning of the liver (normal levels of AST and ALT) and kidneys (with creatinine clearance above 60 mL per minute). To ensure that the participants formed a homogenous group, we only included patients with between three and six months of trastuzumab treatment in the trial. 

We excluded women from the study if they were HER2-negative BC patients or had distant metastases or disease progression that necessitated the introduction of radiation treatment or chemotherapy, cardiac diseases resulting in circulation failure (above the second class of the NYHA), an autoimmune disease (systemic lupus erythematosus, rheumatoid arthritis, Sjogren’s syndrome, etc.), metabolic diseases (obesity, insulin resistance, or type 1 and type 2 diabetes), infectious diseases (human immunodeficiency virus (HIV) or hepatitis C virus (HCV)), chronic inflammatory diseases, lung diseases (chronic obstructive pulmonary disease), or uncontrolled asthma. We eliminated patients who had heart failure progression causing a significant decrease in LVEF below 10% during the oncological observation or under 55% of the total value, and a resting oxygen saturation (SaO_2_) ≤ 92%. Another contraindication was malnutrition (with a body mass index below 18 kg/m^2^), overweight (a BMI above 24.9 kg/m^2^), or weight loss of >10% during the previous three months. Additionally, we ruled out patients with psychiatric or cognitive disorders which made it impossible for them to comply with the standard study recommendations, those who were pregnant (or breastfeeding). The study excluded patients with periodontal disease, smokers, alcohol drinkers, or patients taking antibiotics, non-steroidal anti-inflammatory drugs, corticosteroids, vitamins, or dietary supplements in the previous three months. Furthermore, a participant was excluded if she was being treated with an angiotensin-receptor blocker, an angiotensin-converting-enzyme inhibitor (ACE), or a beta-blocker. 

We carried out observations of BC patients undergoing trastuzumab therapy in two breast cancer groups and in healthy women (control group).

#### 2.2.1. BC + PE Group 

Twenty-five women with BC performed physical exercise training during trastuzumab therapy as an intervention (BC + PE). Our physical training program was based on endurance and strength exercises and aimed at assessing the impact of physical exercise on the negative results of oncological treatment observed before the initiation of training. This training took place on an outpatient rehabilitation ward. The physical activity was moderate, with a maximum heart rate (HRmax) of 70% (according to the calculation HRmax = 220–age) during the exercises [18]. The exercise training for the intervention group consisted of five exercise sessions per week for six weeks. The endurance training involved choosing a maximum of two forms of physical activity for a session from several options: brisk walking, running on a treadmill, and various cycling activities. This part of the daily training program lasted for roughly 60 min according to the schedule: 5 min of warm-up, 50 min of one or two aerobic activities, and 5 min of relaxation time. Additionally, women in the BC + PE group performed strength exercises to build up their muscle mass and increase their strength. This part of the daily session (five days per week) lasted for approximately 60 min. The resistance exercise sessions based on isometric, concentric, and eccentric training consisted of one to four sets of 8–10 repetitions of selected exercises in different positions for the torso, upper body, and leg muscles. To ensure the progressive development of strength, we used different tools in our program: elastic resistance bands (1.5, 2, 2.5, 3, 3.5, and 4 kg per 100% extension), adjustable dumbbells, and medicine balls. The weight training program intensity was progressively raised by increasing the extent of the movement, introducing more heavy exercises, or shifting the velocity of the concentric performance [17,18,19]. Women in this group were instructed not to participate in any other forms of weight exercise during the study time. Their compliance was verified via a daily interview with a physiotherapist who conducted the training. The exercise program was implemented in groups (exercises on treadmills or cycle ergometers, supervised by a therapist) and individually (strength training with the assistance of a physiotherapist). No more than two days were recommended as a maximum break from the training in the course of the study. Participation in this exercise program was verified through a rehabilitation card that was checked by a research physiatrist after the end of the study (after six weeks of training).

#### 2.2.2. Usual Physical Activity Group (without Regular Physical Exercises)

The BC group (N = 25) consisted of women with BC who undertook daily activity according to the cancer care recommendations during trastuzumab therapy [18]. Clinicians provided medical clearance before the patients became involved in the study. The patients in this group were given standard physical activity recommendations and were instructed via printed materials to perform 30 min of moderate physical activity five days/week for 2.5 h/week. 

#### 2.2.3. Control Group 

The control group involved healthy women without cancer or other diseases who performed 2.5 h per week of daily activity, which was verified using a daily activity notebook. The women in this group were instructed not to begin any formal physical activities and, instead, to engage in their usual daily activities at home. 

For the control group (CG), we matched the study group by age, and physical parameters, recruiting generally healthy women using invitation posters.

### 2.3. Methods

The HER2-positive women with BC included in our program were also assessed for changes resulting from trastuzumab therapy. These assessments were performed alongside those for heart failure and physical fitness. 

#### 2.3.1. Capacity Test 

Functional capacity was measured using the 6 min walk test (6MWT) protocol, which is used in many clinical exercise trials to estimate aerobic capacity in cancer patients [20]. This was followed by a 5 min cooldown period conducted in a corridor (30 m). Secondary measures included dyspnea after the test using a modified Borg scale (0–10) and metabolic equivalents (METs). This test can be used as a predictor of functional (distance—6MWD) and objective (VO_2_max) fitness [21]. The MET was calculated as a result of evidence suggesting that 3.5 mL/kg/min does not accurately represent the resting metabolic rate of a general population, standardized METs = (VO_2_/3.5 mL/kg/min) and measured METs = VO_2_/pretest metabolic rate. The pretest metabolic rate was deduced as the mean VO_2_ in the minute prior to commencing the test. Gas analysis (Oxycon Mobile, CareFusion, Germany) was then used.

#### 2.3.2. Doppler Echocardiography

Cardiac function was assessed by a transthoracic Doppler echocardiography according to the European Association of Echocardiography and the American Society of Echocardiography (ASE) recommendations [22] with a Philips echocardiography machine (Philips EPIQ7, Philips Healthcare, Andover, MA, USA) and a 2.5 MHz probe. All echocardiographs were carried out by the same cardiologist. Measurements were performed on 3 representative beats and the average results were recorded. Standard echocardiographic analysis included two-dimensional, M-mode, and Doppler flow measurements. LVEF was assessed in the apical 4- and 2-chamber views using Simpson’s biplane rule; global longitudinal strain as measured by speckle-tracking 2-dimensional echocardiography (in which a negative value is normal, but to allow for easier interpretation, values were presented as positive in this paper); left atrial volume indexed to body surface area; right ventricular function as measured by the tricuspid annular plane systolic excursion; blood pressure and heart ratio. Doppler-derived LV diastolic inflow was performed to measure E and A peak velocities and their ratio E/A.

#### 2.3.3. Redox Measurements

All the reagents (unless otherwise stated) were purchased from Sigma-Aldrich (Nümbrecht, Germany/Saint Louis, MO, USA). The total protein content was measured using the bicinchoninic acid method (Thermo Scientific PIERCE BCA Protein Assay Kit, Rockford, IL, USA), according to the manufacturer’s instructions. The 96-well microplate reader BioTek Synergy H1 (Winooski, VT, USA) was used to measure the samples’ absorbance or fluorescence. All the investigations were conducted with duplicate samples and standardized to 1 mg of total protein.

##### Antioxidant Defenses

The superoxide dismutase (SOD, EC 1.15.1.1) activity was measured spectrophotometrically. One unit of SOD activity was defined as the amount of the enzyme required to inhibit the rate of the reduction in cytochrome c by 50% [23].

The catalase (CAT, EC 1.11.1.6) activity was measured based on H_2_O_2_ decomposition by analyzing the decrease in absorbance at 240 nm. One unit of CAT activity was determined as the quantity of enzyme degrading 1 μmol of H_2_O_2_ in 1 min [24].

The glutathione peroxidase (GSH-Px, EC 1.11.1.9) activity was measured with a colorimetric assay based on the oxidation of NADPH to NADP+. The absorbance was measured at 340 nm. One unit of GPx activity catalyzed the conversion of 1 mmol of NADPH in 1 min [25].

The reduced glutathione (GSH) level was measured colorimetrically using Ellman’s method with 5,5′-dithio-bis-(2-nitrobenzoic acid) (DTNB) [25,26,27]. The absorbance was measured at 412 nm. 

##### Redox Status

The total antioxidant capacity (TAC) was measured using 2,2′-azinobis(3-ethylbenzo-thiazoline-6-sulfonate) (ABTS^*+^), which was decolorized by the antioxidants contained in the test sample. The color change was measured at 660 nm [28]. The total oxidant status (TOS) was measured colorimetrically. Oxidants from the sample transmute the ferrous ion–o-dianisidine complex to ferric ion, forming a colored complex with xylenol orange (XO). The color intensity is proportional to the total quantity of oxidants in the sample [29]. The oxidative stress index (OSI) was calculated as the TOS-to-TAC ratio: OSI = [TOS]/[TAC] [29,30,31].

##### Oxidative Stress

The total lipid hydroperoxide (LOOH) was measured colorimetrically at 560 nm based on the reaction of XO with Fe^3+^ (resulting from Fe^2+^ after its oxidation by LOOH) [32,33].

The advanced oxidation protein product (AOPP) level was measured using a spectrophotometric method at a 340 nm wavelength. The AOPP level corresponds to the rate of the protein oxidation of samples [34].

##### Nitrosative Stress

The total nitric oxide (NO) was measured using a colorimetric assay using sulfanilamide and N-(1-naphthyl)-ethylenediamine dihydrochloride. The absorbance of the reaction product was measured at 490 nm [35,36].

The peroxynitrite level was measured spectrophotometrically based on peroxynitrite-mediated nitration and the production of nitrophenol. The absorbance was measured at a 320 nm wavelength [37].

The S-nitrosothiol level was measured by a fluorometric assay based on the conversion of 2,3-diaminonaphthylene to 2,3-naphthotriazole by S-nitrosothiol compounds [38].

The nitrotyrosine level was measured by an enzyme-linked immunosorbent assay (ELISA). A commercially available kit (Nitrotyrosine ELISA; Immundiagnostik AG, Bensheim, Germany) was used, following the manufacturer’s instructions.

#### 2.3.4. Statistical Analysis

GraphPad Prism 8.3.4 for macOS (GraphPad Software, Inc., La Jolla, CA, USA) was used for statistical data processing. The statistical significance level was set at *p* < 0.05. The normality of the distribution was assessed using the Shapiro–Wilk test, while the homogeneity of the variance was checked with Levene’s test. A one-way Kruskal–Wallis analysis of variance (ANOVA) followed by Dunn’s post hoc test was used to compare the quantitative variables. Multiplicity-adjusted *p*-values were also calculated.

The number of patients in the group was determined based on our previous experiment, assuming that the power of the test = 0.9 and α = 0.05.

## 3. Results

### 3.1. Study Participants

Our analysis of the study participants did not reveal important differences between their physical parameters or medical history. The characteristics the enrolled study patients are presented in Table 1. 

### 3.2. Capacity Test

Analysis of the physical activity performance of the participants did not reveal important differences between the study groups. The results of the physical capacity tests for the study participants are shown in Table 2. 

### 3.3. The Analysis of Echocardiography Results

The analysis of echocardiography results of cardiac function in study groups is presented in Table 3. The echocardiography parameters did not differ statistically significantly between study groups. 

### 3.4. Differences in Antioxidant Enzymes (SOD, CAT, and GSH-Px) and Non-Enzymatic Antioxidants (GSH) in the Study Groups

**Figure 1:**
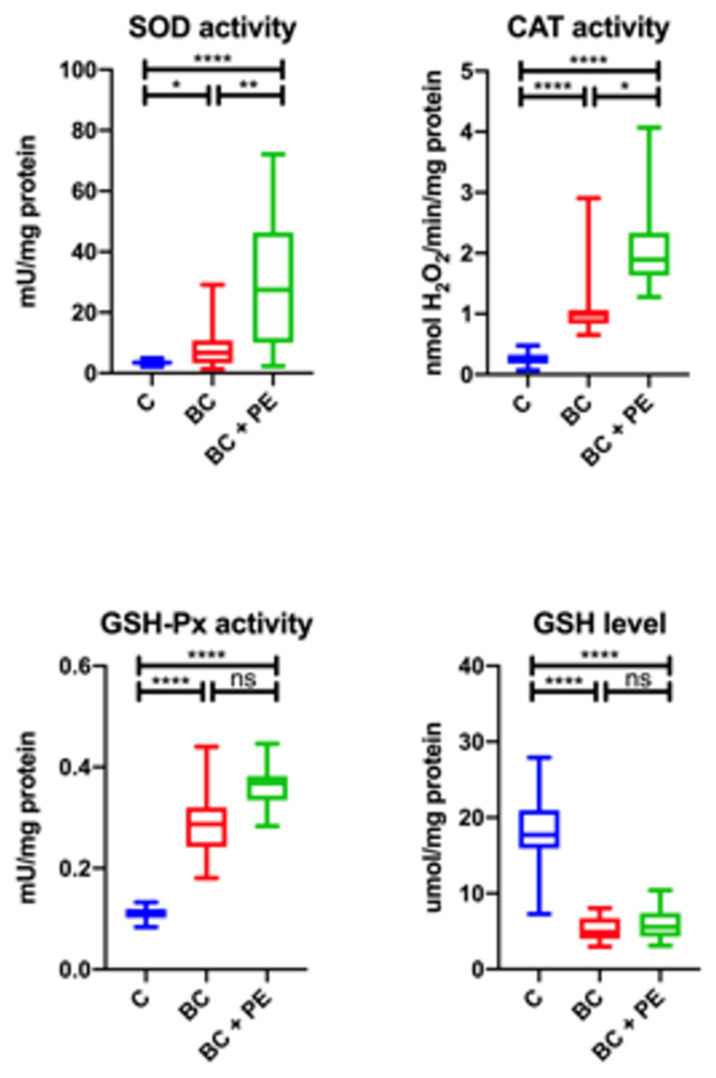
Results for antioxidant enzymes (SOD, CAT, and GSH-Px) and non-enzymatic antioxidants (GSH) in the study groups. * *p* < 0.05, ** *p* < 0.01, *** *p* < 0.001, **** *p* < 0.0001, ns-not statistically important.

We evaluated the antioxidant defenses according to antioxidant enzymes (SOD, CAT, and GSH-Px) and non-enzymatic antioxidants (GSH). The SOD and CAT activities were significantly higher in the BC + PE group than the BC and control groups. The GSH-Px activity concentration were significantly higher in both study groups than in the healthy controls. The GSH activities was significantly higher in healthy controls. The results for the differences in the antioxidant enzymes (SOD, CAT, and GSH-Px) and non-enzymatic antioxidants (GSH) in the study groups are presented in Figure 1.

### 3.5. Analysis of Results for Redox Status of Study Groups

**Figure 2:**
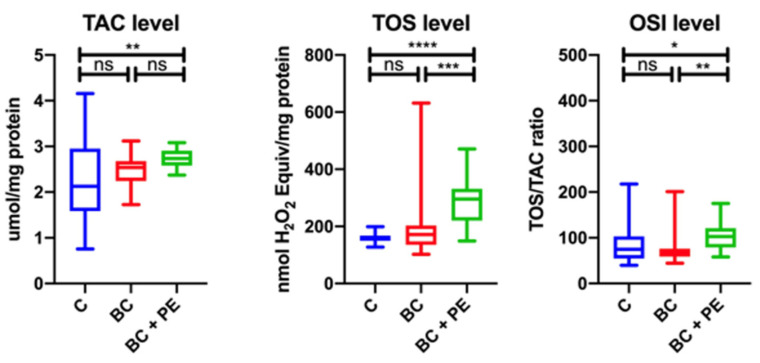
Analysis of results for redox status of the study groups. * *p* < 0.05, ** *p* < 0.01, *** *p* < 0.001, **** *p* < 0.0001, ns-not statistically important.

The TAC, TOS and OSI levels were significantly higher in the BC + PE group compared with the BC group and healthy controls. The results for those parameters are shown in Figure 2. 

### 3.6. Analysis of Results for Oxidative Stress of Study Groups

**Figure 3:**
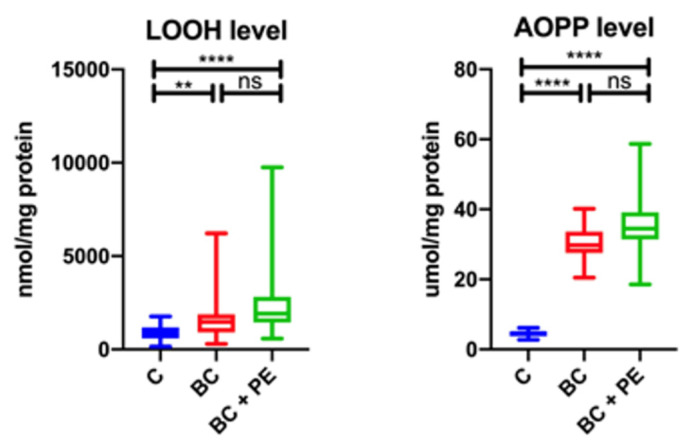
Comparison of results for LOOH and AOPP levels between the study groups. * *p* < 0.05, ** *p* < 0.01, *** *p* < 0.001, **** *p* < 0.0001, ns-not statistically important.

The concentrations of lipid (LOOH) and protein (AOPP) oxidative damage products were significantly higher in both the BC study groups than in the healthy controls. A comparison of the results for the LOOH and AOPP levels between the study groups is shown in Figure 3. 

### 3.7. Analysis of Results for Nitrosative Stress Parameters of Study Groups 

**Figure 4:**
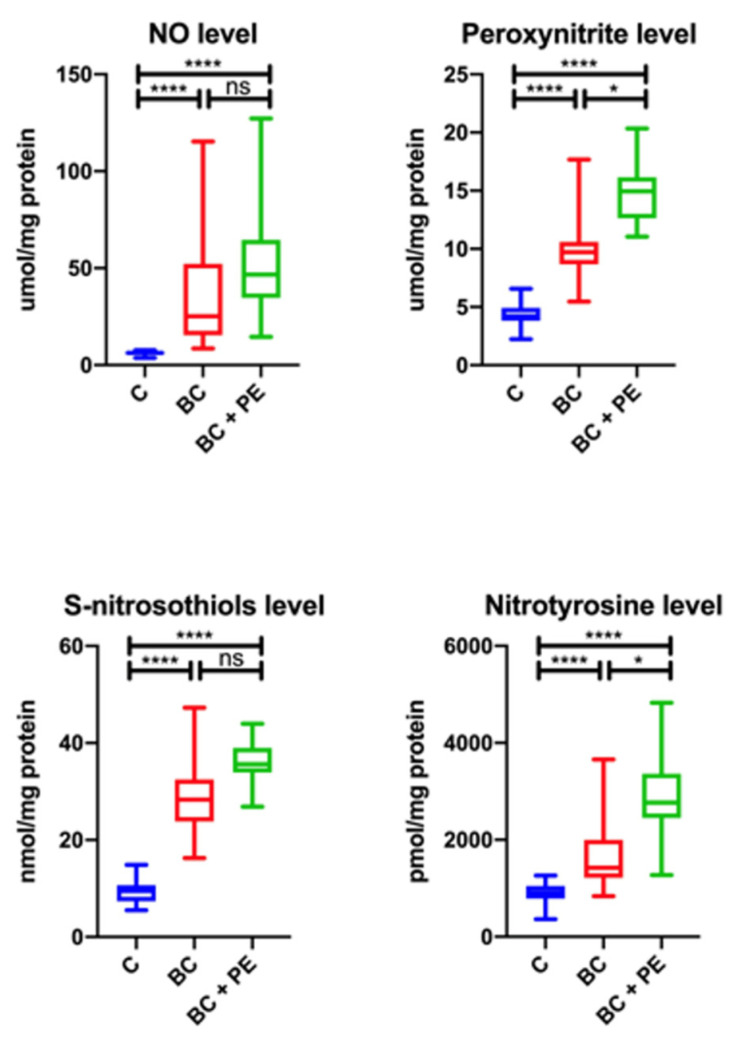
Results for nitrosative stress parameters in the study groups. * *p* < 0.05, ** *p* < 0.01, *** *p* < 0.001, **** *p* < 0.0001, ns-not statistically important.

The concentrations of total NO and S-nitrosothiols were significantly higher in the BC and BC + PE groups than in the controls (Figure 4). The peroxynitrite and nitrotyrosine levels were statistically significantly higher in the BC + PE group than in the cancer patients without regular physical activity and the controls.

## 4. Discussion 

Many epidemiological studies indicate that diet and physical activity may reduce individuals’ cancer risk and improve their prognosis [17,18]. In women with BC, exercise training has been shown to reduce the risk of cancer death by 20–50%, especially when recreational activities are practiced [39]. Physical activity is important in post-mastectomy women because exercise rehabilitation reduces the risk of upper limb swelling and improves shoulder mobility [18,39]. However, little is known about the effects of physical activity in breast cancer patients treated with trastuzumab [40,41,42,43]. Trastuzumab is a monoclonal antibody selectively directed against the HER2 receptor, the over expression/of which occurs in about a quarter of total BC cases [1]. Unfortunately, the drug can cause side effects that mainly involve the cardiovascular system [2,3]. Trastuzumab has been shown to induce oxidative-stress-mediated cardiotoxicity [7,8]; however, its effects on systemic redox homeostasis are not well understood, nor are the effects of physical exercise. Therefore, we evaluated selected biomarkers of oxidative and nitrosative stress in trastuzumab-treated BC patients performing regular moderate-intensity exercise, compared with those in patients with routine physical activity and healthy subjects.

Trastuzumab works by attaching itself to the HER2 receptors and can help in fighting BC by alerting the immune system to destroy cancer cells to which it is attached. As a previous study documented [2,3], the use of trastuzumab can potentiate cardiomyocyte damage through a ‘dual-hit’ mechanism, which includes the inhibition of the neuregulin-1 survival signaling pathway and angiotensin-II-induced activation of NADPH oxidase (NOX), with the further ability to increase ROS production [2,3,5]. The blockade of HER2 thereby leads to ROS accumulation within the cardiomyocytes, which may lead to the development of cardiac dysfunction by stimulating cardiomyocyte apoptosis. The various mechanisms of trastuzumab-induced cardiotoxicity may also include antioxidant-related oxidative stress. The advancement of heart failure could be connected to an imbalance between ROS production and antioxidative defenses, which leads to oxidative damage to proteins, lipids, and nucleic acids, causing structural damage to cells and disturbances in tissue integrity [8,44]. When ROS production occurs in the absence of sufficient defense mechanisms, several genomic modifications may arise, including chromosomal instability, DNA damage, and the acquisition of mutations, which could contribute to the development of carcinogenesis [45].

It is well-documented that regular exercise training can improve the physical fitness and capacity of patients by enhancing the antioxidative status of the body [14,15,16,46]. In our study, different biomarkers were used to assess systemic redox homeostasis, e.g., the enzymatic (SOD, CAT, and Px) and non-enzymatic (GSH) antioxidant systems, total redox status (TAC, TOS, and OSI), and protein (AOPP) and lipid (LOOP) oxidative damage products. In women with BC undergoing trastuzumab therapy, we showed a significant increase in the activity of antioxidant enzymes (SOD, CAT, and GSH-Px) vs. the control group (healthy women), which may be an adaptive response of the body to the intensified production of free radicals. It is well-known that strengthening the antioxidant barrier is one of the primary strategies for protecting against cellular/systemic oxidative stress. Similar observations were reported by Tomasello et al. [47] and Carter et al. [48]. However, the non-enzymatic antioxidant agent (reduced glutathione, GSH) was significantly lower in the BC groups. GSH acts as the primary thiol buffer of the cell, to maintain the oxidoreductive state. GSH is also involved in repairing proteins, lipids, and DNA and the regeneration of other antioxidants such as vitamins C and E. This compound also regulates the expression of receptors for protein kinases and transcription factors such as NF-κB (nuclear factor kappa-light-chain-enhancer of activated B cells), the stimulation of which significantly enhances ROS production. Not surprisingly, despite the enhancement of the enzymatic antioxidant defenses, oxidative damage to proteins and lipids occurs in BC patients, which may be due to the depletion of glutathione reserves. Indeed, according to our observations, the concentrations of lipid (LOOH) and protein (AOPP) oxidative damage products were significantly higher in both BC groups than in the healthy controls. Analysis of the total redox status (TOS, TAC, and OSI) also confirmed previous observations. The TOS and TAC provide more information than evaluating redox parameters separately. It is well-known that TOS and TAC characterize the resultant capacity for ROS generation/scavenging in biological systems. The oxidative stress index, i.e., the ratio of oxidative (TOS) over antioxidative (TAC) processes, was significantly higher in BC patients who exercised regularly and moderately compared with those BC patients undertaking routine daily activity and the healthy controls. Exercise training leads to increased oxidative stress, although this same stimulus is necessary to allow an upregulation of endogenous antioxidant defenses [46,47,49]. Indeed, with the oxidation of energy substrates in mitochondria, excessive ROS formation occurs. Since the ROS production depends on the intensity and duration of exercise, as well as the individual’s level of training [50,51], it is not surprising that oxidative stress is greater in BC patients who exercise regularly. The concentrations of nitrosative stress biomarkers (NO and S-nitrosothiols) were also significantly higher in the BC groups than in the healthy controls. Lemos et al. [44] observed similar results in BC patients undergoing trastuzumab-based chemotherapy with BC during chemotherapy without trastuzumab treatment and healthy controls, but those authors did not observed patients during supervised physical exercise intervention. In our results, the peroxynitrite and nitrotyrosine levels were higher in the BC patients during trastuzumab treatment who exercised regularly compared with cancer women during trastuzumab therapy without regular physical activity or healthy controls. We found higher levels peroxynitrite and nitrotyrosine in BC patients during trastuzumab treatment the same as in Lemos et al. study [44]. Their findings showed increased NO levels in BC during trastuzumab treatment than that in either BC during chemotherapy without trastuzumab or healthy controls. Furthermore, data also revealed that AOPP levels were significantly higher in the BC during trastuzumab therapy than the control group. Increased chronic nitrosative and oxidative stress is dangerous for the body since it leads to a loss of the integrity and stability of biological structures, and thus, to permanent abnormalities in their metabolism [31,36,50]. As such, implementing moderate intensity physical activity may not reduce the cardiovascular complications of trastuzumab by reducing oxidative and nitrosative stress. Additionally clinical trials are needed to analyze the effects of personalized, different intensities and levels of physical training in BC women who are undergoing trastuzumab treatment. Although further studies are needed, supplementation with antioxidants, especially those that enhance endogenous GSH production, may be advisable to consider for BC patients treated with trastuzumab.

Limitations and Strengths

One of the limitations in our study was the small number of study participants; therefore, this was a feasibility study. The study observation time was only carried out one time; thus, further observations are required. However, this study has some strengths. Firstly, our study was a one of first clinical, controlled trial where authors analyzed BC during trastuzumab treatment in aspect of implementation of the physical exercise training and redox response. Secondly, our technical staff were blinded to the group allocation and blood measurements of the participants. Finally, the exercise protocol was standardized by strict parameters for the volume of training sessions (heart rate, repetitions, sets, etc.). 

## 5. Conclusions

In conclusion, the results presented in this paper highlight the oxidative imbalance in parallel with regular physical training in women with BC during trastuzumab therapy. Regular moderate-intensity exercise was not sufficient to reverse oxidative damage in this group of patients. Those results are important in aspect of cardiac dysfunctions which are common during trastuzumab therapy. Further studies are needed to analyze the effects of different intensities and levels of physical training in women with BC who are undergoing trastuzumab treatment.

## Figures and Tables

**Table 1:** Patients’ characteristics.

Parameter		CG (N = 50)	BC (N = 25)	BC + PE (N = 25)	*p*
Age [years]	mean ± SD	54.64 ± 4.26	53.43 ± 3.68	52.24 ± 7.29	0.78
Weight [kg]	mean ± SD	68.73 ± 7.5	63.25 ± 5.53	65.69 ± 8.51	0.121
Height [m]	mean ± SD	1.68 ± 0.57	1.65 ± 0.64	1.66 ± 0.24	0.234
BMI [kg/m^2^]	mean ± SD	24.22 ± 2.46	25.35 ± 1.89	24.35 ± 5.6	0.409
**Medical history N (%)**					
Surgical treatment	BCT	0	13 (52%)	11 (44%)	
	Mastectomy	0	2 (8%)	3 (12%)	
	Mastectomy with reconstruction	0	10 (40%)	11 (44%)	
Side of operated breast cancer	Left	0	14 (56%)	16 (64%)	
	Right	0	11 (44%)	9 (36%)	
Cancer stage	IB	0	1 (4%)	2 (8%)	
	IIA	0	11 (44%)	9 (36%)	
	IIB	0	13 (52%)	14 (56%)	
Previous anthracycline treatment		0	20 (80%)	22(88%)	
Hormonal therapy		0	22 (88%)	22(88%)	
NYHA I		13(26%)	9 (36%)	13 (26%)	
NYHA II		2(4%)	4 (16%)	2 (4%)	
**Another illness bearing a cardiac risk N (%)**					
Coronary artery disease		0 (0%)	0 (0%)	0 (0%)	
Dyslipidemia		2 (4%)	3 (12%)	4 (16%)	
Hypertension		3 (6%)	2 (8%)	5 (20%)	
History of smoking		2 (4%)	1 (%)	2 (8%)	

Abbreviations: SD—standard deviation; *p*—statistical significance; BC—breast cancer group; BC + PE—breast cancer group who regularly performed physical exercise; CG—control group; BMI—body mass index; BCT—breast-conserving therapy; NYHA—New York Heart Association.

**Table 2:** Physical capacity test results for the study participants.

Parameters	CG (N = 50)	BC (N = 25)	BC + PE (N = 25)	*p*
	Mean ± SD			
6MWD [m]	451.6 ± 55.33	441.6 ± 24.88	449.7 ± 50.06	0.314
MET Unit	3.19 ± 0.26	3.11 ± 0.12	3.14 ± 0.24	0.211
Borg scale [point]	1.29 ± 0.7	1.82 ± 0.6	1.62 ± 0.72	0.698

Abbreviations: SD—standard deviation; m—meters; BC—breast cancer group; BC + PE—breast cancer group who regularly performed physical exercise; CG—control group; 6MWD—six-minute walking distance; MET—metabolic equivalent; *p*—statistical significance.

**Table 3:** Results of the cardiac function assessment between study groups.

Parameters	CG (N = 50)	BC (N = 25)	BC + PE (N = 25)	*p*
	Mean ± SD			
LVEF [%]	65.59 ± 5.02	63,9 ± 2.52	64.46 ± 3.71	0.114
GLS [%]	17.4 ± 2.5	16.9 ± 2.5	16.8 ± 2.5	0.271
LAVI [mL/m^2^]	24.5 ± 2.5	23.8 ± 2.5	24.2 ± 2.5	0.177
RVEF [%]	53.2 ± 4.5	52.5 ± 7.1	52.2 ± 6.4	0.563
TAPSE [mm]	21.1 ± 3.1	20.1 ± 3.6	20.4 ± 2.7	0.662
E/A	1.5 ± 0.5	1.4 ± 0.5	1.4 ± 0.5	0.878

Abbreviations: BC—breast cancer group; BC + PE—breast cancer group who regularly performed physical exercise; CG—control group; LVEF-left ventricular ejection fraction; GLS-global longitudinal strain (for ease of interpretation, a measure of GLS having negative values, here uses positive values); LAVI-left atrial volume index; RVEF-right ventricular ejection fraction; TAPSE- tricuspid annular plane systolic excursion; SD—standard deviation; *p*—statistical significance.

## Data Availability

The datasets used and/or analyzed during this study are stored in a permanent repository and are available from the corresponding author on reasonable request.
